# Development and internal validation of an interpretable machine learning model using first-24-h postpartum nursing variables to predict delayed lactogenesis II after cesarean delivery

**DOI:** 10.3389/fmed.2026.1892993

**Published:** 2026-08-10

**Authors:** Tianzhi Guo, Yuqin Sun

**Affiliations:** 1 Department of Obstetrics, The Affiliated Hospital, Southwest Medical University, Luzhou, China; 2 Department of Oral and Maxillofacial Surgery, The Affiliated Hospital, Southwest Medical University, Luzhou, China

**Keywords:** breastfeeding, cesarean delivery, delayed lactogenesis II, machine learning, SHAP, XGBoost

## Abstract

**Background:**

Delayed lactogenesis II is a common early postpartum breastfeeding problem after cesarean delivery and may interfere with the establishment of exclusive breastfeeding. Although demographic and obstetric risk factors have been widely studied, the predictive value of routinely recorded early postpartum nursing process variables remains insufficiently explored. This study aimed to develop and internally validate a dynamic 24-h postpartum landmark interpretable machine learning model using clinical, perioperative, neonatal, and first-24-h nursing process variables to predict delayed lactogenesis II by 72 h after cesarean delivery.

**Methods:**

This single-center retrospective cohort study included women who underwent cesarean delivery at a tertiary hospital between January 2021 and December 2024 and had documented intention to breastfeed. Delayed lactogenesis II was defined as the absence of standardized evidence of lactogenesis onset by 72 h postpartum, based on maternal responses and supporting indicators documented by trained nurses at predefined postpartum assessments. The prediction landmark was set at 24 h postpartum; therefore, only variables available before delivery, during cesarean delivery, or within the first 24 h postpartum were used for model development. Because women who had already experienced lactogenesis II by 24 h were no longer at risk at the prediction landmark, an additional sensitivity analysis was restricted to women without lactogenesis II onset by the 24-h assessment. Candidate predictors were extracted from electronic medical records, perioperative records, neonatal records, nursing assessment documentation, and breastfeeding assessment forms. A complete-case approach was used; 32 women (2.2% of those initially screened) were excluded because of incomplete key predictor data, and no statistical imputation was performed. The final cohort was randomly divided into a training cohort and an internal validation cohort at a ratio of 7:3. Five models were developed and compared: logistic regression, random forest, support vector machine (SVM), XGBoost, and LightGBM. Hyperparameters were selected within the training cohort using repeated stratified five-fold cross-validation, and the internal validation cohort remained untouched until final model evaluation. Model performance was assessed using the area under the receiver operating characteristic curve, accuracy, sensitivity, specificity, positive predictive value, negative predictive value, and *F*1 score. Calibration curve analysis, decision curve analysis, and SHAP-based interpretation were performed for the final model.

**Results:**

A total of 1,286 women were included, of whom 326 developed delayed lactogenesis II, corresponding to an event rate of 25.3%. Among the included women, 71 (5.5%) had already experienced lactogenesis II by the 24-h assessment and had been retained in the primary cohort. The training and validation cohorts included 900 and 386 women, respectively. In the validation cohort, all five models showed acceptable discrimination, with AUCs ranging from 0.816 to 0.836. The support vector machine achieved the highest validation AUC of 0.836, followed by LightGBM, XGBoost, random forest, and logistic regression. XGBoost showed a training AUC of 0.872 and a validation AUC of 0.823, with validation accuracy, sensitivity, specificity, positive predictive value, negative predictive value, and *F*1 score of 0.756, 0.694, 0.778, 0.515, 0.882, and 0.591, respectively. Although SVM achieved the numerically highest validation AUC, its discrimination did not differ significantly from that of XGBoost (AUC difference, 0.013; 95% *CI*, −0.019 to 0.045; DeLong *P* = 0.421). XGBoost was retained as the final interpretable model because it showed a smaller training-to-validation AUC difference and enabled direct SHAP-based characterization of predictor contributions. In the internal validation cohort, the XGBoost model had a calibration intercept of 0.058, a calibration slope of 1.684 (95% *CI*, 1.304–2.064), and a Brier score of 0.143. Although the intercept indicated limited systematic miscalibration, the calibration slope and its confidence interval were entirely above the ideal value of 1, indicating non-ideal calibration and under-dispersion of predicted probabilities, with predictions compressed toward the mean. Decision curve analysis indicated greater net benefit than the treat-all and treat-none strategies across clinically relevant threshold probabilities. SHAP analysis identified first breastfeeding time, breastfeeding frequency within 24 h, first skin-to-skin contact time, intraoperative blood loss, and postoperative pain score at 24 h as the most influential predictors. After excluding the 71 women with lactogenesis II onset by 24 h, the landmark-restricted sensitivity cohort comprised 1,215 women. In this restricted population, the XGBoost model achieved a validation AUC of 0.817, and the five leading predictors remained unchanged.

**Conclusions:**

A dynamic 24-h postpartum landmark interpretable machine learning model incorporating clinical, perioperative, neonatal, and first-24-h nursing process variables showed acceptable discrimination for predicting delayed lactogenesis II by 72 h after cesarean delivery, but its calibration was non-ideal. Although SVM achieved the numerically highest validation AUC, its discrimination did not differ significantly from that of XGBoost. XGBoost was retained as the final interpretable model on the basis of its overall balance of validation discrimination, apparent stability, and direct SHAP-based interpretability. First breastfeeding time, breastfeeding frequency, skin-to-skin contact, intraoperative blood loss, and postoperative pain score may serve as early postpartum risk markers to help identify women who could benefit from intensified breastfeeding support and individualized nursing care. Because the model has undergone only single-center internal validation, external validation and recalibration are required before it can be used to estimate individual risk or support individual-level clinical decision-making.

## Introduction

Breastfeeding is a fundamental component of early maternal and infant health care. It provides optimal nutrition for infants, supports immune protection and neurodevelopment, and is associated with both short-term and long-term health benefits for mothers and children (1, 2). Beyond its biological benefits, breastfeeding is increasingly recognized as a public health priority requiring coordinated support across maternity care, family, community, and health-system levels (3, 4). In clinical practice, however, the successful establishment of breastfeeding depends not only on maternal intention but also on early postpartum physiological transition, mother–infant contact, professional support, and timely management of breastfeeding difficulties (5).

Lactogenesis II, also referred to as secretory activation, marks the transition from colostrum secretion to copious milk production and usually occurs within the first 2–3 days after delivery. Delayed lactogenesis II is commonly defined as the absence of clear maternal perception or clinical evidence of milk “coming in” beyond 72 h postpartum (6, 7). This condition is clinically important because delayed milk production may increase maternal anxiety about insufficient milk supply, promote early formula supplementation, impair exclusive breastfeeding, and contribute to early breastfeeding discontinuation (8). Therefore, early identification of women at increased risk of delayed lactogenesis II may allow maternity nurses and clinicians to provide timely lactation support before breastfeeding difficulties become established.

Cesarean delivery represents a particularly important clinical context for studying delayed lactogenesis II. Compared with vaginal birth, cesarean delivery may delay mother–infant contact, postpone the first breastfeeding attempt, reduce early breastfeeding frequency, and increase maternal postoperative pain and fatigue (9). Women undergoing cesarean delivery may also experience delayed ambulation, difficulty with breastfeeding positioning, and interruptions in rooming-in or neonatal contact because of perioperative routines or neonatal observation. Previous reviews have suggested that women who deliver by cesarean section are more likely to experience delayed breastfeeding initiation and early breastfeeding difficulties, particularly when breastfeeding support is insufficient (10).

The onset of lactogenesis II is influenced by multiple maternal, obstetric, neonatal, and care-process factors. Earlier studies have identified higher maternal body mass index, primiparity, cesarean delivery, prolonged labor, delayed breastfeeding initiation, and ineffective early infant suckling as potential risk factors for delayed lactogenesis (11). Maternal overweight or obesity may affect lactation through hormonal, metabolic, inflammatory, and mechanical pathways, whereas primiparity may reflect limited breastfeeding experience and a greater need for practical nursing support (12). However, many previous studies have focused mainly on demographic or obstetric predictors, while routinely recorded nursing process variables have received less attention.

In postpartum nursing practice, variables such as first skin-to-skin contact time, first breastfeeding time, breastfeeding frequency within the first 24 h, sleep duration during the first postpartum night, and postoperative pain score are routinely documented and clinically meaningful. These variables may reflect both the physiological stimulation required for milk production and the quality of early breastfeeding support after cesarean delivery. Unlike many baseline demographic characteristics, several nursing process variables are potentially modifiable and may therefore serve not only as predictors but also as actionable targets for early intervention. Incorporating these variables into prediction models may improve the clinical relevance of risk stratification and help nurses identify women who require intensified lactation support.

Clinical prediction models provide a structured approach for estimating individualized risk and supporting targeted care. Traditional regression-based models are interpretable and widely used, but they may have limited ability to capture non-linear relationships and complex interactions among clinical, perioperative, neonatal, and nursing variables. Machine learning methods may complement conventional statistical approaches by modeling more flexible patterns in structured clinical data (13). Nevertheless, machine learning models are often criticized as “black boxes,” especially when used in clinical decision-making. Therefore, for postpartum nursing applications, model interpretability is essential to ensure that predicted risk can be translated into understandable and actionable clinical strategies.

To date, limited evidence is available on interpretable machine learning models that incorporate routinely collected early postpartum nursing assessment data to predict delayed lactogenesis II after cesarean delivery. This gap is clinically important because delayed lactogenesis II is common, early postpartum nursing assessment is feasible, and several care-process variables within the first 24 h after delivery may be modifiable. Therefore, this study aimed to develop and internally validate a dynamic 24-h postpartum landmark interpretable machine learning model using clinical, perioperative, neonatal, and first-24-h nursing process variables to predict delayed lactogenesis II by 72 h postpartum after cesarean delivery. SHAP-based interpretation was further applied to identify the most influential predictors and clarify their contributions to predicted risk, with the goal of supporting individualized early postpartum risk reassessment and targeted breastfeeding support.

## Methods

### Study design

This single-center retrospective cohort study was conducted to develop and internally validate a dynamic 24-h postpartum landmark interpretable machine learning model for predicting delayed lactogenesis II by 72 h after cesarean delivery. The model was designed as an early postpartum dynamic risk reassessment tool rather than a preoperative, immediate post-delivery, or causal model. The prediction landmark was prespecified at 24 h postpartum. Candidate predictors were therefore restricted to information documented before or at the 24-h landmark, whereas the outcome was determined according to whether standardized evidence of lactogenesis II had occurred by 72 h postpartum. This temporal separation was used to ensure that information recorded after the prediction landmark was not incorporated into model development. This study was reported in accordance with the TRIPOD+AI statement for clinical prediction models developed or evaluated using regression or machine learning methods (14).

The overall workflow included retrospective cohort screening, data extraction, variable organization, outcome-stratified random cohort allocation, repeated cross-validation within the training cohort, model development, independent holdout validation, calibration assessment, decision curve analysis, and SHAP-based model interpretation. Repeated cross-validation was used for hyperparameter selection, whereas the internal validation cohort was held out for final performance evaluation.

### Setting

The study was conducted in the Department of Obstetrics of a tertiary hospital. Consecutive women who underwent cesarean delivery between January 2021 and December 2024 were retrospectively screened. Clinical, perioperative, neonatal, and nursing assessment data were obtained from the electronic medical record system, anesthesia and operative records, neonatal records, postpartum nursing documentation, and breastfeeding assessment forms. All data were collected as part of routine clinical care and were extracted retrospectively for research purposes.

### Participants

Women were eligible if they underwent cesarean delivery during the study period, had documented intention to breastfeed after delivery, and had available clinical and nursing assessment information within the first 24 h postpartum. The exclusion criteria were as follows: no breastfeeding intention, severe psychiatric illness or cognitive impairment that could affect breastfeeding assessment, serious maternal systemic disease affecting postpartum lactation evaluation, neonatal death or major congenital anomaly, missing outcome data, or missing key predictor data required for model development.

Among the 1,472 women initially screened, 186 were excluded: 72 had no documented intention to breastfeed, 11 had severe psychiatric illness or cognitive impairment, 29 had serious maternal systemic disease affecting lactation assessment, 33 had neonatal death or a major congenital anomaly, 9 had missing outcome data, and 32 had missing data for at least one key predictor required for model development. The latter 32 women represented 2.2% of the initially screened cohort. A complete-case analysis was therefore performed, and no statistical imputation was used. The final complete-case analytic cohort comprised 1,286 women, who were subsequently allocated at a ratio of 7:3 to the training cohort (*n* = 900) and internal validation cohort (*n* = 386) using outcome-stratified random sampling to preserve the proportion of women with delayed lactogenesis II in both cohorts. Among the 1,286 women included in the primary analysis, 71 (5.5%) had documented onset of lactogenesis II by the 24-h assessment, including 49 women in the training cohort and 22 women in the internal validation cohort. These women were included in the primary analysis because the outcome was initially operationalized according to whether lactogenesis II had occurred by 72 h postpartum, and all 71 women were classified as not having delayed lactogenesis II. However, because they were no longer at risk of delayed lactogenesis II at the 24-h prediction landmark, an additional landmark-restricted sensitivity analysis was performed after excluding them.

### Outcome definition and standardized assessment

The primary outcome was delayed lactogenesis II by 72 h postpartum. As part of routine postpartum breastfeeding care, lactogenesis onset was assessed at approximately 24, 48, and 72 h after delivery using a standardized nursing assessment form. At the first assessment, the mother was asked: “Since delivery, have you noticed that your breasts have become noticeably fuller, heavier, or firmer, together with a clear increase in the amount of milk produced?” At the subsequent 48- and 72-h assessments, the mother was asked: “Since the previous assessment, have you noticed that your breasts have become noticeably fuller, heavier, or firmer, together with a clear increase in the amount of milk produced?” Nurses documented the maternal response and supporting indicators, including a clear maternal perception that milk had “come in,” increased breast fullness or heaviness, spontaneous milk leakage, and an appreciable increase in expressed or transferred milk compared with the preceding assessment. Lactogenesis II onset was assigned to the earliest postpartum assessment at which the mother reported a clear increase in milk production accompanied by at least one documented supporting indicator. Delayed lactogenesis II was defined as the absence of this standardized evidence of lactogenesis onset by 72 h postpartum.

Because the prediction landmark was set at 24 h postpartum, the intended target population comprised women who had not yet experienced lactogenesis II and therefore remained at risk of delayed lactogenesis II at the 24-h assessment. In the primary analysis, however, women with documented lactogenesis II onset by 24 h were retained because the outcome was initially operationalized according to lactogenesis status by 72 h postpartum. Information obtained during the 48- and 72-h lactogenesis assessments was used only to determine the outcome and was not used to derive, select, or transform any predictor. Variables recorded after the 24-h landmark or directly reflecting subsequent lactogenesis status were excluded from model development to preserve temporal separation between predictors and outcome-related information. To examine the influence of including women who were no longer at risk at the landmark, an additional sensitivity analysis was restricted to women without documented lactogenesis II onset by 24 h.

### Candidate predictors

Candidate predictors were selected according to clinical relevance, availability in routine obstetric and nursing records, and availability no later than 24 h postpartum. The model used information available before delivery, during cesarean delivery, and within the first 24 h postpartum to estimate the risk of delayed lactogenesis II by 72 h postpartum.

Candidate predictors included demographic characteristics, obstetric variables, perioperative factors, neonatal characteristics, and first-24-h postpartum nursing process variables. Demographic and obstetric variables included admission year, maternal age, pre-pregnancy body mass index, body mass index at delivery, gestational age, primiparity, previous cesarean delivery, gestational diabetes mellitus, hypertensive disorders of pregnancy, elective cesarean delivery, and cesarean indication.

Perioperative variables included neuraxial anesthesia, operation time, intraoperative blood loss, and postoperative pain score at 24 h. Neonatal variables included neonatal intensive care unit admission, birth weight, and 5-min Apgar score. First-24-h postpartum nursing process variables included first skin-to-skin contact time, first breastfeeding time, breastfeeding frequency within 24 h, and sleep duration during the first postpartum night. Breast engorgement by 48 h and formula supplementation within 72 h were considered outcome-proximal postpartum indicators because they were recorded after the 24-h prediction landmark and could reflect emerging or established lactation difficulty. These variables were presented only for descriptive clinical characterization and were excluded from all prediction models, hyperparameter tuning, calibration assessment, decision curve analysis, and SHAP interpretation to reduce the risk of information leakage.

### Data sources and measurement

Data were extracted from the hospital electronic medical record system, anesthesia and operative records, obstetric nursing records, neonatal records, and breastfeeding assessment forms. Maternal age, obstetric history, pregnancy complications, delivery information, and neonatal characteristics were obtained from standardized electronic records. Operation time and intraoperative blood loss were extracted from operative and anesthesia records. Postoperative pain score at 24 h was recorded by nurses using the routine numerical rating scale. Lactogenesis assessments were scheduled at approximately 24, 48, and 72 h postpartum and were recorded in predefined fields of the standardized breastfeeding assessment form. The recorded information included the assessment time, the maternal response to the standardized question, and the presence or absence of each supporting indicator.

The recorded information included the maternal response to the standardized lactogenesis question, the assessment time, and the presence or absence of supporting indicators. The same operational criteria were applied throughout the study period. During retrospective extraction, the 72-h outcome was assigned according to a prespecified data dictionary. Records with unclear or internally inconsistent documentation were independently reviewed by two investigators, and disagreements were resolved by consensus. All predictors used for model development were recorded no later than 24 h postpartum. Variables recorded after the 24-h prediction landmark or variables directly reflecting lactogenesis status by 72 h were not used as predictors.

Continuous variables were retained on their original scale where appropriate. Categorical variables were coded according to clinical definitions. Binary variables were coded as 0 for absence and 1 for presence. Multicategory variables, including admission year, cesarean indication, and 5-min Apgar score, were transformed into dummy variables for machine learning model development.

### Missing data

The extent of missingness was assessed before cohort allocation and model development. Among the 1,472 women initially screened, 9 (0.6%) had missing 72-h lactogenesis outcome data and were excluded because their outcome status could not be determined. A further 32 women (2.2% of the initially screened cohort) had missing data for at least one key candidate predictor required for model development. Thus, the participant-level proportion with incomplete key predictor information was 2.2%. Because this proportion was small, the primary analysis used a complete-case approach, and no single or multiple imputation was performed. The final analytic cohort comprised 1,286 complete cases, with no residual missing values in the outcome or candidate predictors used for modeling. The complete-case cohort was established before outcome-stratified allocation into the training and internal validation cohorts, and the same complete predictor set was used across all five modeling approaches. No model-specific exclusion or imputation was performed after cohort allocation. A summary of missing-data completeness is provided in Supplementary Table 5.

### Bias control

Several measures were implemented to reduce potential bias. First, consecutive women who underwent cesarean delivery during the study period were screened to minimize selection bias. Second, predictors were obtained from routinely collected clinical, perioperative, neonatal, and nursing records rather than from *post hoc* maternal recall, thereby reducing recall bias. The individuals responsible for retrospective data extraction were not formally blinded to the 72-h lactogenesis outcome. To reduce the potential influence of outcome knowledge on predictor ascertainment, all candidate predictors were defined in advance using a prespecified data dictionary and were extracted primarily from structured or time-stamped source fields. Predictor eligibility was determined solely by whether the information had been documented by the prespecified 24-h postpartum landmark, without reinterpretation based on subsequent lactogenesis status. Third, lactogenesis onset was assessed using the same departmental breastfeeding assessment form, standardized assessment time points, and predefined operational criteria throughout the study period. Obstetric nurses received standardized orientation and routine competency training in postpartum breastfeeding assessment. During retrospective outcome extraction, unclear or internally inconsistent records were independently reviewed by two investigators and resolved by consensus according to the predefined outcome criteria to reduce outcome misclassification. Finally, information obtained from the 48- and 72-h lactogenesis assessments was used exclusively to determine the outcome and was not used to define or reinterpret the first-24-h predictors. Variables recorded after the 24-h landmark or closely related to subsequent lactogenesis status, including breast engorgement by 48 h and formula supplementation within 72 h, were excluded from preprocessing, hyperparameter tuning, model fitting, calibration assessment, decision curve analysis, and SHAP interpretation. These procedures maintained temporal separation between predictor ascertainment and outcome determination and reduced, although could not completely eliminate, the risks of information leakage and observer-related information bias.

### Study size

The study size was determined by the number of eligible women who underwent cesarean delivery during the study period and met the inclusion and exclusion criteria. A total of 1,286 women were included, of whom 326 developed delayed lactogenesis II. The overall event rate was 25.3%. The training cohort included 900 women, including 228 events, and the validation cohort included 386 women, including 98 events. This sample size provided an adequate number of outcome events for model development, internal validation, and comparison of multiple machine learning algorithms.

### Quantitative variables

Continuous variables were summarized as median and interquartile range because several clinical and nursing variables were not normally distributed. Categorical variables were summarized as frequencies and percentages. Continuous predictors, including maternal age, body mass index, gestational age, operation time, intraoperative blood loss, postoperative pain score, first skin-to-skin contact time, first breastfeeding time, breastfeeding frequency within 24 h, sleep duration, and birth weight, were analyzed on their original continuous scale. No data-driven categorization of continuous predictors was performed, in order to preserve information and reduce loss of statistical power.

For model development, categorical predictors were converted into dummy variables. Continuous predictors were standardized for SVM using parameters estimated from the corresponding training resample, whereas standardization was not required for logistic regression or the tree-based models. To prevent information leakage, all preprocessing parameters were estimated within each cross-validation training fold and then applied to its corresponding assessment fold. After hyperparameter selection, the preprocessing procedure was re-estimated using the complete training cohort and applied unchanged to the internal validation cohort.

### Statistical analysis

All statistical analyses were performed using R software, version 4.4.1 (R Foundation for Statistical Computing, Vienna, Austria). Missingness was assessed before cohort allocation and model development. The primary analysis used a complete-case approach; women with missing outcome data or missing data for at least one key predictor required for model development were excluded, and no statistical imputation was performed. Random allocation into the training and internal validation cohorts was conducted only after the complete-case analytic cohort had been established. Baseline characteristics were compared between the training and validation cohorts to assess the balance of random allocation. In the training cohort, clinical and nursing characteristics were further compared according to delayed lactogenesis II status. Multicollinearity was assessed in the training cohort before model fitting. Pairwise relationships between continuous predictors were examined using Spearman and Pearson correlation coefficients, and variance inflation factors (VIFs) were calculated for the predictor matrix. A *VIF* ≥ 5 was considered indicative of potentially important collinearity, whereas a *VIF* ≥ 10 was considered indicative of severe multicollinearity. Because the study objective was prediction rather than estimation of independent causal effects, correlated predictors were not automatically excluded solely on the basis of the VIF; their clinical relevance, temporal meaning, and potential influence on model interpretation were also considered. Continuous variables were compared using the Wilcoxon rank-sum test, and categorical variables were compared using Pearson's chi-square test or Fisher's exact test, as appropriate. All statistical tests were two-sided, and a *P*-value <0.05 was considered statistically significant.

The modeling task was defined as a 24-h postpartum landmark prediction task. Predictors available up to 24 h postpartum were used to estimate the risk of delayed lactogenesis II by 72 h postpartum. The complete-case cohort was allocated at a ratio of 7:3 to a training cohort and an internal validation cohort using outcome-stratified random sampling with a fixed random seed of 2026. Stratification preserved the proportion of delayed lactogenesis II events in both cohorts. The training cohort was used for preprocessing, hyperparameter tuning, model fitting, and threshold selection, whereas the internal validation cohort was held out and evaluated only after all model-development decisions had been completed. This independent holdout cohort was used for the final assessment of discrimination, calibration, and classification performance.

Five prediction models were developed and compared: logistic regression, random forest, support vector machine (SVM), XGBoost, and LightGBM. Logistic regression was included as an unpenalized conventional reference model because it is a well-established probabilistic classification method with directly interpretable coefficients and remains widely used in medical prediction research (15). Previous research in obstetric and neonatal settings has also demonstrated the value of comparing conventional multivariable statistical methods with machine learning classifiers when developing clinical prediction models (16). Logistic regression did not require hyperparameter tuning in the present analysis.

Hyperparameter tuning for the other four algorithms was performed exclusively within the training cohort using repeated stratified five-fold cross-validation with five repetitions. Stratification preserved the proportion of women with delayed lactogenesis II in each fold. For each candidate parameter combination, the mean cross-validated AUC across the resamples was calculated, and the combination with the highest mean AUC was selected. Dummy-variable encoding and algorithm-specific feature standardization were performed within each resampling iteration to prevent information leakage. After selection of the optimal hyperparameters, each model was refitted using the complete training cohort and evaluated once in the untouched internal validation cohort. The parameter search ranges and final selected values are reported in Supplementary Table 1. The optimal classification threshold for each model was determined in the training cohort using the Youden index and was then applied unchanged to the internal validation cohort. No information from the internal validation cohort was used for data preprocessing, hyperparameter selection, model fitting, or threshold determination. A random seed of 2026 was used to ensure reproducibility.

Model discrimination was evaluated using the area under the receiver operating characteristic curve. Classification performance was assessed using accuracy, sensitivity, specificity, positive predictive value, negative predictive value, and *F*1 score. ROC curves were plotted for both the training and validation cohorts. Because all models were evaluated in the same internal validation cohort, the validation AUCs of SVM and XGBoost were formally compared using a paired DeLong test for correlated ROC curves. The absolute AUC difference, its 95% confidence interval, and the corresponding two-sided *P*-value were reported. A *P*-value <0.05 was considered statistically significant. Calibration of the final XGBoost model was evaluated in the internal validation cohort using a calibration plot, calibration intercept, calibration slope, and Brier score. The calibration intercept was estimated by fitting a logistic recalibration model with the model linear predictor included as an offset and its coefficient fixed at 1; an intercept of 0 indicates the absence of systematic overestimation or underestimation of average risk. The calibration slope was estimated by regressing the observed outcome on the logit of the predicted probability; a slope of 1 indicates ideal spread of the predicted risks, a slope below 1 suggests overly extreme predictions, and a slope above 1 suggests under-dispersed predictions. The Brier score was calculated as the mean squared difference between the predicted probabilities and observed binary outcomes, with lower values indicating better overall probabilistic accuracy. Clinical utility was evaluated using decision curve analysis by comparing the net benefit of the final model with treat-all and treat-none strategies across clinically relevant threshold probabilities.

To provide an illustrative example of potential clinical implementation, an exploratory risk-stratification analysis was performed in the internal validation cohort. Women were categorized according to their XGBoost-predicted probability as low risk (<0.15), moderate risk (0.15 to <0.30), or high risk (≥0.30). The number of women and the observed incidence of delayed lactogenesis II were calculated for each group, and differences in event proportions were assessed using Pearson's chi-square test. These categories were intended as an exploratory clinical illustration and were not treated as validated intervention thresholds.

A landmark-restricted sensitivity analysis was performed after excluding women who had documented onset of lactogenesis II by the 24-h assessment, because these women were no longer at risk of delayed lactogenesis II at the prediction landmark. To preserve comparability with the primary analysis, the original training and internal validation assignments were retained. The complete modeling procedure was repeated in the restricted population using the same preprocessing, hyperparameter-tuning, model-fitting, threshold-selection, and validation procedures as in the primary analysis. Discrimination, calibration, and SHAP-based predictor importance were reassessed in the restricted at-risk population.

The final model was selected by considering validation discrimination, the statistical comparison of AUCs, the magnitude of the training-to-validation AUC difference, and the feasibility of clinically interpretable model explanation. Although SVM achieved the numerically highest validation AUC, its AUC did not differ significantly from that of XGBoost according to the paired DeLong test. XGBoost was therefore retained as the final interpretable model because it combined statistically comparable validation discrimination with a smaller training-to-validation AUC difference and enabled direct SHAP-based assessment of global and individual-level predictor contributions. SHAP values were calculated in the validation cohort for the final XGBoost model. Predictors were ranked according to the mean absolute SHAP value, and SHAP summary plots were used to visualize the direction and magnitude of each predictor's contribution to the predicted risk of delayed lactogenesis II.

## Results

### Baseline characteristics of the training and validation cohorts

**Figure 1:**
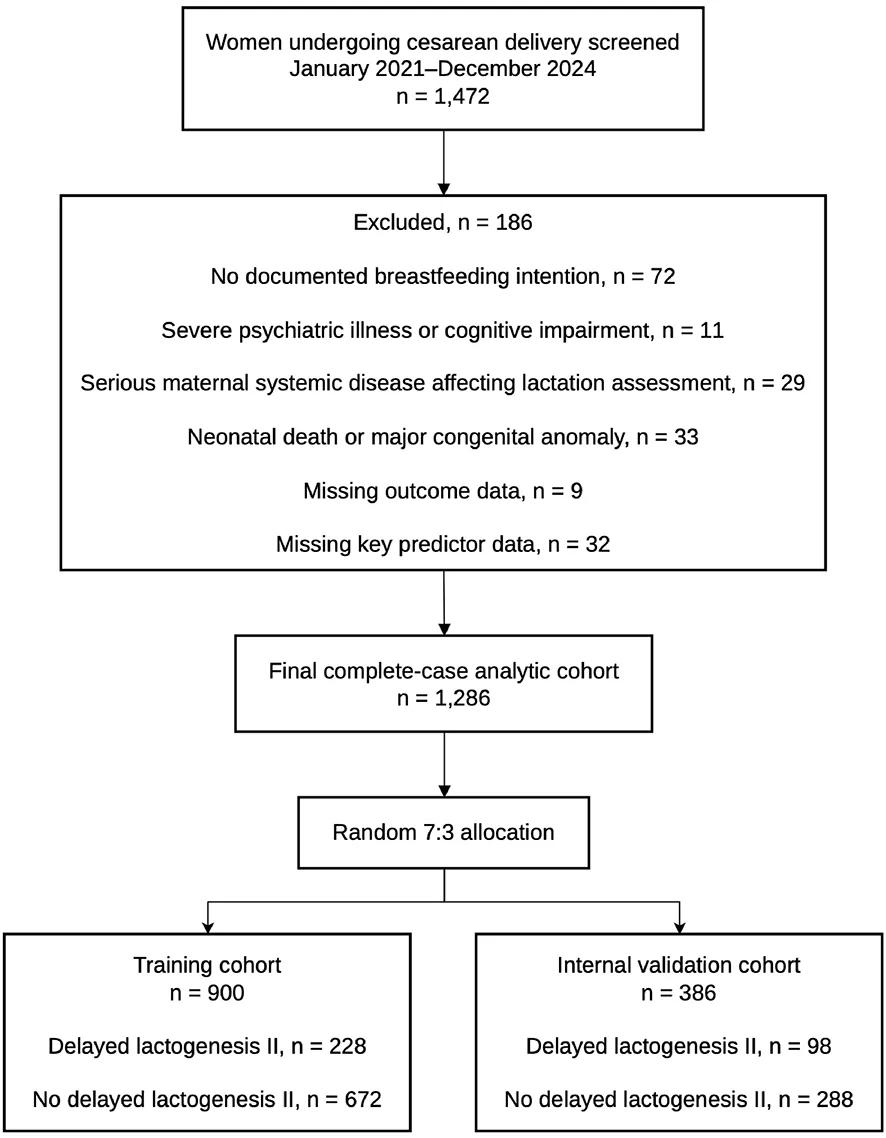
Flowchart of participant selection and model-development framework. Women who underwent cesarean delivery between January 2021 and December 2024 were retrospectively screened. After application of the eligibility criteria, the final complete-case cohort comprised 1,286 women. Outcome-stratified random sampling was used to allocate women to the training cohort (*n* = 900; 228 events) and internal validation cohort (*n* = 386; 98 events) at a ratio of 7:3. Repeated stratified five-fold cross-validation with five repetitions was performed within the training cohort for hyperparameter selection. After tuning, each model was refitted using the complete training cohort and evaluated once in the untouched internal validation cohort. The final XGBoost model underwent calibration assessment, decision curve analysis, and SHAP-based interpretation. SHAP, SHapley Additive exPlanations; XGBoost, extreme gradient boosting.

A total of 1,472 women who underwent cesarean delivery were initially screened. Of these, 9 (0.6%) were excluded because of missing outcome data and 32 (2.2%) because of missing data for at least one key candidate predictor required for model development. After application of all eligibility criteria, the final complete-case analytic cohort comprised 1,286 women, with no residual missing values in the outcome or candidate predictors used for modeling (Supplementary Table 5). The cohort was allocated using outcome-stratified random sampling to a training cohort of 900 women and an internal validation cohort of 386 women (Figure 1). The training and internal validation cohorts included 228 and 98 women with delayed lactogenesis II, corresponding to event rates of 25.3% and 25.4%, respectively. Of the 1,286 women included in the primary analysis, 71 (5.5%) had documented onset of lactogenesis II by the 24-h assessment, including 49 women in the training cohort and 22 women in the internal validation cohort. All 71 women were classified as not having delayed lactogenesis II by 72 h postpartum. The overall median maternal age was 31.0 years, and the median gestational age was 38.6 weeks. The median pre-pregnancy BMI and BMI at delivery were 23.6 kg/m^2^ and 29.1 kg/m^2^, respectively. Overall, 50.0% of women were primiparous, 27.6% had a previous cesarean delivery, 15.0% had gestational diabetes mellitus, and 8.0% had hypertensive disorders of pregnancy. Elective cesarean delivery accounted for 49.8% of cases. The median operation time was 56.0 min, and the median intraoperative blood loss was 286.0 ml. Regarding first-24-h postpartum nursing process variables, the median first skin-to-skin contact time was 50.0 min, the median first breastfeeding time was 5.9 h, and the median breastfeeding frequency within 24 h was 5.0 times. No statistically significant differences were observed between the training and validation cohorts in demographic characteristics, obstetric factors, perioperative variables, neonatal characteristics, or first-24-h postpartum nursing indicators, indicating that the two cohorts were well balanced after random allocation (all *P* > 0.05; Table 1).

**Table 1:** Baseline characteristics of the training and validation cohorts.

Characteristic	Overall (*n* = 1,286)	Training cohort (*n* = 900)	Validation cohort (*n* = 386)	*P*-value
Admission year				0.741
2021	307 (23.9%)	221 (24.6%)	86 (22.3%)	
2022	343 (26.7%)	242 (26.9%)	101 (26.2%)	
2023	310 (24.1%)	215 (23.9%)	95 (24.6%)	
2024	326 (25.3%)	222 (24.7%)	104 (26.9%)	
Maternal age, years	31.0 (28.0, 34.0)	31.0 (28.0, 34.0)	31.0 (28.0, 34.0)	0.142
Pre-pregnancy BMI, kg/m^2^	23.6 (21.3, 25.8)	23.6 (21.2, 25.8)	23.7 (21.5, 25.8)	0.480
BMI at delivery, kg/m^2^	29.1 (26.7, 31.5)	29.0 (26.6, 31.4)	29.3 (27.0, 31.6)	0.301
Gestational age, weeks	38.6 (37.9, 39.3)	38.6 (37.9, 39.4)	38.5 (38.0, 39.2)	0.766
Primipara	643 (50.0%)	449 (49.9%)	194 (50.3%)	0.951
Previous cesarean delivery	355 (27.6%)	254 (28.2%)	101 (26.2%)	0.491
Gestational diabetes mellitus	193 (15.0%)	132 (14.7%)	61 (15.8%)	0.662
Hypertensive disorder of pregnancy	103 (8.0%)	68 (7.6%)	35 (9.1%)	0.422
Elective cesarean delivery	641 (49.8%)	454 (50.4%)	187 (48.4%)	0.551
Cesarean indication				0.885
Fetal distress	172 (13.4%)	117 (13.0%)	55 (14.2%)	
GDM-related indication	68 (5.3%)	46 (5.1%)	22 (5.7%)	
Hypertensive disorder	104 (8.1%)	69 (7.7%)	35 (9.1%)	
Malpresentation	155 (12.1%)	107 (11.9%)	48 (12.4%)	
Maternal request	173 (13.5%)	124 (13.8%)	49 (12.7%)	
Placenta/other	98 (7.6%)	67 (7.4%)	31 (8.0%)	
Previous cesarean	357 (27.8%)	260 (28.9%)	97 (25.1%)	
Suspected macrosomia	159 (12.4%)	110 (12.2%)	49 (12.7%)	
Neuraxial anesthesia	1,104 (85.8%)	779 (86.6%)	325 (84.2%)	0.305
Operation time, min	56.0 (47.0, 64.0)	56.0 (47.0, 64.0)	55.0 (47.0, 65.0)	0.777
Intraoperative blood loss, ml	286.0 (210.0, 380.0)	283.0 (206.5, 380.0)	290.0 (214.0, 380.0)	0.298
Postoperative pain score at 24 h	4.0 (3.0, 5.0)	4.0 (3.0, 5.0)	4.0 (3.0, 5.0)	0.303
First skin-to-skin contact time, min	50.0 (35.0, 70.0)	50.0 (35.0, 69.5)	50.5 (35.0, 73.0)	0.684
First breastfeeding time, h	5.9 (4.0, 8.3)	5.8 (4.0, 8.4)	6.4 (4.1, 8.2)	0.409
Breastfeeding frequency within 24 h	5.0 (4.0, 7.0)	5.0 (4.0, 7.0)	5.0 (4.0, 7.0)	0.414
Sleep duration during the first night, h	3.9 (3.1, 4.9)	3.9 (3.1, 4.9)	4.0 (3.2, 5.0)	0.166
Neonatal NICU admission	95 (7.4%)	64 (7.1%)	31 (8.0%)	0.644
Birth weight, g	3,263.0 (3,010.0, 3,522.0)	3,268.5 (3,024.0, 3,535.0)	3,256.0 (3,002.0, 3,495.0)	0.272
Five-min Apgar score				0.875
7	3 (0.2%)	2 (0.2%)	1 (0.3%)	
8	109 (8.5%)	73 (8.1%)	36 (9.3%)	
9	579 (45.0%)	410 (45.6%)	169 (43.8%)	
10	595 (46.3%)	415 (46.1%)	180 (46.6%)	
Breast engorgement by 48 h	706 (54.9%)	486 (54.0%)	220 (57.0%)	0.353
Formula supplementation within 72 h	428 (33.3%)	288 (32.0%)	140 (36.3%)	0.154

Continuous variables are presented as median (interquartile range), and categorical variables are presented as *n* (%). *P*-values were calculated using the Wilcoxon rank-sum test for continuous variables and Pearson's chi-square test for categorical variables. BMI, body mass index; GDM, gestational diabetes mellitus; NICU, neonatal intensive care unit.

### Clinical and nursing characteristics according to delayed lactogenesis II status

In the training cohort, 228 of 900 women developed delayed lactogenesis II by 72 h postpartum. Compared with women without delayed lactogenesis II, those with delayed lactogenesis II had higher pre-pregnancy BMI [24.3 (22.1, 26.4) vs. 23.3 (21.0, 25.6) kg/m^2^, *P* < 0.001] and higher BMI at delivery [29.9 (27.3, 32.3) vs. 28.6 (26.4, 31.2) kg/m^2^, *P* < 0.001]. The delayed lactogenesis II group also had higher proportions of primiparity (58.8% vs. 46.9%, *P* = 0.002), hypertensive disorders of pregnancy (14.0% vs. 5.4%, *P* < 0.001), and elective cesarean delivery (59.2% vs. 47.5%, *P* = 0.003). Regarding perioperative and first-24-h postpartum nursing process variables, women with delayed lactogenesis II had greater intraoperative blood loss [334.0 (245.0, 446.0) vs. 269.0 (194.5, 350.0) ml, *P* < 0.001], higher postoperative pain scores at 24 h [4.0 (3.0, 6.0) vs. 4.0 (3.0, 5.0), *P* < 0.001], delayed first skin-to-skin contact [65.0 (44.0, 102.0) vs. 46.5 (32.5, 63.5) min, *P* < 0.001], later first breastfeeding [8.5 (5.8, 10.5) vs. 5.2 (3.7, 7.2) h, *P* < 0.001], and fewer breastfeeding episodes within 24 h [4.0 (3.0, 5.0) vs. 6.0 (4.0, 7.0), *P* < 0.001]. Neonatal NICU admission was also more frequent in the delayed lactogenesis II group (19.3% vs. 3.0%, *P* < 0.001), and birth weight was slightly higher [3,335.0 (3,095.5, 3,581.5) vs. 3,243.5 (3,002.5, 3,519.5) g, *P* = 0.007].

In contrast, maternal age, gestational age, previous cesarean delivery, cesarean indication, neuraxial anesthesia, operation time, sleep duration during the first postpartum night, and 5-min Apgar score did not differ significantly between groups. Breast engorgement by 48 h was less common, whereas formula supplementation within 72 h was more common among women with delayed lactogenesis II (both *P* < 0.001; Table 2). These outcome-proximal postpartum indicators were described for clinical characterization only and were not used as predictors in model development.

**Table 2:** Clinical and nursing characteristics according to delayed lactogenesis II status in the training cohort.

Characteristic	Overall (*n* = 900)	No delayed lactogenesis II (*n* = 672)	Delayed lactogenesis II (*n* = 228)	*P*-value
Admission year				0.958
2021	221 (24.6%)	166 (24.7%)	55 (24.1%)	
2022	242 (26.9%)	178 (26.5%)	64 (28.1%)	
2023	215 (23.9%)	160 (23.8%)	55 (24.1%)	
2024	222 (24.7%)	168 (25.0%)	54 (23.7%)	
Maternal age, years	31.0 (28.0, 34.0)	31.0 (28.0, 34.0)	31.0 (28.5, 34.0)	0.459
Pre-pregnancy BMI, kg/m^2^	23.6 (21.2, 25.8)	23.3 (21.0, 25.6)	24.3 (22.1, 26.4)	<0.001
BMI at delivery, kg/m^2^	29.0 (26.6, 31.4)	28.6 (26.4, 31.2)	29.9 (27.3, 32.3)	<0.001
Gestational age, weeks	38.6 (37.9, 39.4)	38.6 (37.9, 39.4)	38.6 (38.0, 39.3)	0.939
Primipara	449 (49.9%)	315 (46.9%)	134 (58.8%)	0.002
Previous cesarean delivery	254 (28.2%)	198 (29.5%)	56 (24.6%)	0.181
Gestational diabetes mellitus	132 (14.7%)	89 (13.2%)	43 (18.9%)	0.050
Hypertensive disorder of pregnancy	68 (7.6%)	36 (5.4%)	32 (14.0%)	<0.001
Elective cesarean delivery	454 (50.4%)	319 (47.5%)	135 (59.2%)	0.003
Cesarean indication				0.294
Fetal distress	117 (13.0%)	85 (12.6%)	32 (14.0%)	
GDM-related indication	46 (5.1%)	29 (4.3%)	17 (7.5%)	
Hypertensive disorder	69 (7.7%)	47 (7.0%)	22 (9.6%)	
Malpresentation	107 (11.9%)	82 (12.2%)	25 (11.0%)	
Maternal request	124 (13.8%)	91 (13.5%)	33 (14.5%)	
Placenta/other	67 (7.4%)	48 (7.1%)	19 (8.3%)	
Previous cesarean	260 (28.9%)	204 (30.4%)	56 (24.6%)	
Suspected macrosomia	110 (12.2%)	86 (12.8%)	24 (10.5%)	
Neuraxial anesthesia	779 (86.6%)	579 (86.2%)	200 (87.7%)	0.629
Operation time, min	56.0 (47.0, 64.0)	56.0 (47.0, 64.0)	56.0 (47.0, 67.0)	0.309
Intraoperative blood loss, ml	283.0 (206.5, 380.0)	269.0 (194.5, 350.0)	334.0 (245.0, 446.0)	<0.001
Postoperative pain score at 24 h	4.0 (3.0, 5.0)	4.0 (3.0, 5.0)	4.0 (3.0, 6.0)	<0.001
First skin-to-skin contact time, min	50.0 (35.0, 69.5)	46.5 (32.5, 63.5)	65.0 (44.0, 102.0)	<0.001
First breastfeeding time, h	5.8 (4.0, 8.4)	5.2 (3.7, 7.2)	8.5 (5.8, 10.5)	<0.001
Breastfeeding frequency within 24 h	5.0 (4.0, 7.0)	6.0 (4.0, 7.0)	4.0 (3.0, 5.0)	<0.001
Sleep duration during the first night, h	3.9 (3.1, 4.9)	3.9 (3.1, 4.8)	3.8 (3.0, 4.9)	0.375
Neonatal NICU admission	64 (7.1%)	20 (3.0%)	44 (19.3%)	<0.001
Birth weight, g	3,268.5 (3,024.0, 3,535.0)	3,243.5 (3,002.5, 3,519.5)	3,335.0 (3,095.5, 3,581.5)	0.007
Five-min Apgar score				0.181
7	2 (0.2%)	2 (0.3%)	0 (0.0%)	
8	73 (8.1%)	50 (7.4%)	23 (10.1%)	
9	410 (45.6%)	298 (44.3%)	112 (49.1%)	
10	415 (46.1%)	322 (47.9%)	93 (40.8%)	
Breast engorgement by 48 h	486 (54.0%)	418 (62.2%)	68 (29.8%)	<0.001
Formula supplementation within 72 h	288 (32.0%)	151 (22.5%)	137 (60.1%)	<0.001

BMI, body mass index; GDM, gestational diabetes mellitus; NICU, neonatal intensive care unit. Continuous variables are presented as median (interquartile range), and categorical variables are presented as *n* (%). *P*-values were calculated using the Wilcoxon rank-sum test for continuous variables and Pearson's chi-square test for categorical variables. Delayed lactogenesis II was defined as the absence of standardized evidence of lactogenesis onset at the 72-h postpartum assessment, based on maternal responses and supporting indicators recorded on the departmental breastfeeding assessment form.

### Multicollinearity assessment

Multicollinearity assessment showed a strong correlation between pre-pregnancy BMI and BMI at delivery in the training cohort (Spearman's *r*_*s*_ = 0.938, *P* < 0.001; Pearson's *r* = 0.938, *P* < 0.001). The corresponding VIFs were 8.83 for pre-pregnancy BMI and 8.78 for BMI at delivery, indicating substantial but not severe multicollinearity according to the prespecified VIF threshold of 10. The VIFs of the remaining non-redundant continuous predictors ranged from 1.04 to 2.50. Both BMI variables were retained because they represented clinically distinct time points and the primary objective was prediction rather than estimation of independent regression effects. Detailed results of the multicollinearity assessment are provided in Supplementary Table 3.

### Model performance and validation

**Figure 2:**
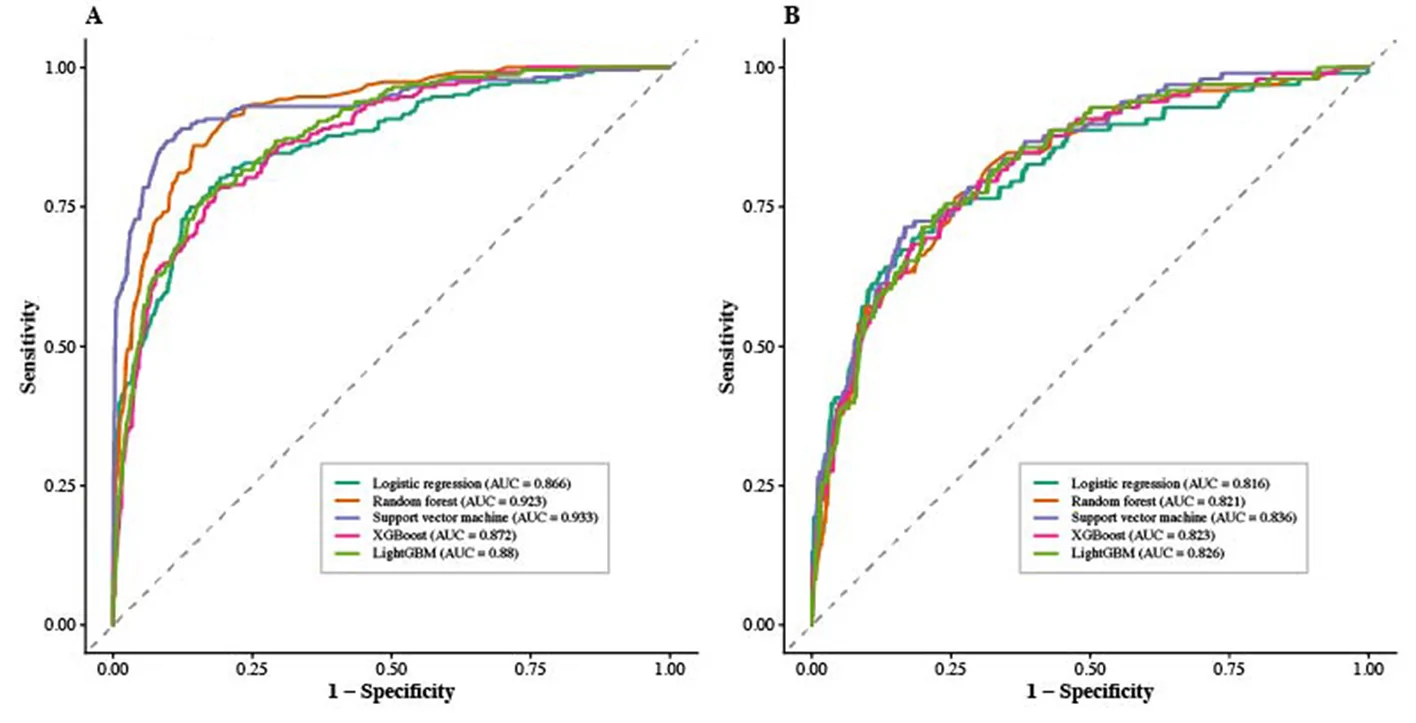
ROC curves of five machine learning models in the training and validation cohorts. ROC curves comparing logistic regression, random forest, support vector machine, XGBoost, and LightGBM models in the training cohort **(A)** and internal validation cohort **(B)**. AUC values are shown in the legends. In the internal validation cohort, the absolute AUC difference between SVM and XGBoost was 0.013 and was not statistically significant according to the paired DeLong test (95% CI, −0.019 to 0.045; *P* = 0.421). AUC, area under the receiver operating characteristic curve; ROC, receiver operating characteristic; SVM, support vector machine; XGBoost, extreme gradient boosting; LightGBM, light gradient boosting machine.

The overall workflow of model development, validation, and interpretation is shown in Figure 1. Five machine learning models were developed in the training cohort to estimate the risk of delayed lactogenesis II by 72 h postpartum using predictors available up to the 24-h postpartum landmark. Hyperparameter tuning was performed within the training cohort using repeated stratified five-fold cross-validation with five repetitions, with the mean cross-validated AUC used to select the optimal parameter combination. The final parameter settings for each model are provided in Supplementary Table 1. After hyperparameter selection, the models were refitted using the complete training cohort and evaluated once in the untouched internal validation cohort. The models were evaluated in both the training cohort and the internal validation cohort. In the validation cohort, all models showed acceptable discrimination, with AUC values ranging from 0.816 to 0.836 (Table 3 and Figure 2).

**Table 3:** Performance comparison of machine learning models in the training and validation cohorts.

Model	Training AUC	Validation AUC	Validation accuracy	Validation sensitivity	Validation specificity	Validation PPV	Validation NPV	Validation *F*1 score	Threshold
Logistic regression	0.866	0.816	0.801	0.653	0.851	0.598	0.878	0.624	0.320
Random forest	0.923	0.821	0.754	0.704	0.771	0.511	0.884	0.592	0.142
Support vector machine	0.933	0.836	0.759	0.724	0.771	0.518	0.892	0.604	0.244
XGBoost	0.872	0.823	0.756	0.694	0.778	0.515	0.882	0.591	0.264
LightGBM	0.880	0.826	0.772	0.653	0.812	0.542	0.873	0.593	0.280

AUC, area under the receiver operating characteristic curve; PPV, positive predictive value; NPV, negative predictive value; *F*1, *F*1 score; XGBoost, extreme gradient boosting; LightGBM, light gradient boosting machine. The optimal threshold for each model was determined in the training cohort using the Youden index and then applied to the validation cohort. Validation accuracy, sensitivity, specificity, PPV, NPV, and *F*1 score were calculated in the validation cohort using the training-derived threshold. Hyperparameter tuning was performed exclusively in the training cohort using repeated stratified five-fold cross-validation with five repetitions. The mean cross-validated AUC was used to select the optimal parameter combination, after which each model was refitted using the complete training cohort and evaluated in the untouched internal validation cohort. Detailed search ranges and final parameter settings are provided in Supplementary Table 1. Quantitative calibration measures for the final XGBoost model, including the calibration intercept, calibration slope, and Brier score, are provided in Supplementary Table 2.

**Figure 3:**
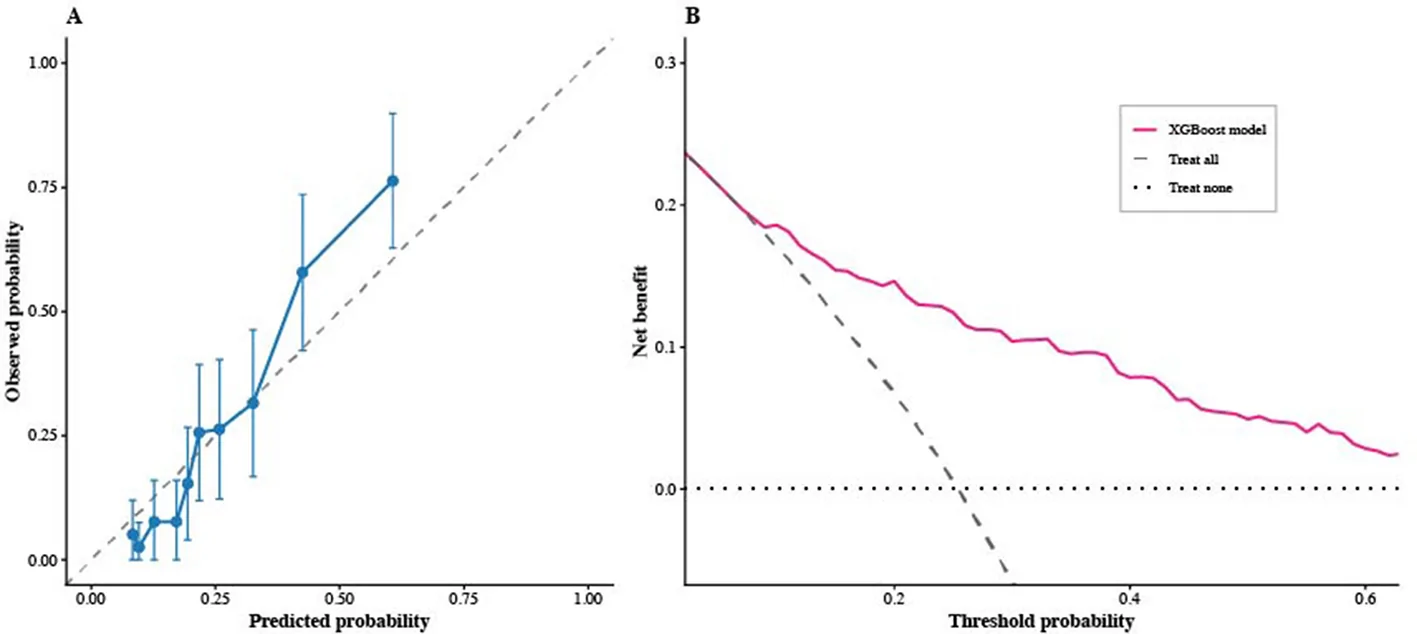
Calibration and decision curve analyses of the final XGBoost model. Calibration plot of the final XGBoost model in the internal validation cohort **(A)** and decision curve analysis across threshold probabilities **(B)**. In **(A)**, the dashed diagonal line represents perfect calibration, and the plotted points represent grouped observed vs. predicted probabilities. Quantitative calibration assessment yielded a calibration intercept of 0.058 (95% CI, −0.187 to 0.303), a calibration slope of 1.684 (95% CI, 1.304 to 2.064), and a Brier score of 0.143. The intercept indicated limited systematic miscalibration. However, the calibration slope and its 95% confidence interval were entirely above the ideal value of 1, indicating non-ideal calibration and under-dispersion of predicted probabilities, with predictions compressed toward the mean. External validation and recalibration are required before these predicted probabilities can be used for individual-level clinical decision-making. In **(B)**, the net benefit of the XGBoost model is compared with treat-all and treat-none strategies. XGBoost, extreme gradient boosting; CI, confidence interval.

The support vector machine achieved the numerically highest validation AUC of 0.836, followed by LightGBM (*AUC* = 0.826), XGBoost (*AUC* = 0.823), random forest (*AUC* = 0.821), and logistic regression (*AUC* = 0.816). In the formal comparison relevant to final model selection, the absolute difference in validation AUC between SVM and XGBoost was 0.013 (95% *CI*, −0.019 to 0.045), which was not statistically significant according to the paired DeLong test (*P* = 0.421). XGBoost demonstrated a training AUC of 0.872 and a validation AUC of 0.823, with validation accuracy, sensitivity, specificity, PPV, NPV, and *F*1 score of 0.756, 0.694, 0.778, 0.515, 0.882, and 0.591, respectively (Table 3). Although SVM achieved the numerically highest validation AUC, the DeLong test did not demonstrate a statistically significant difference between SVM and XGBoost. Moreover, the training-to-validation AUC difference was smaller for XGBoost than for SVM (0.049 vs. 0.097), indicating less apparent performance optimism across the random split. XGBoost was therefore retained as the final interpretable model on the basis of its statistically comparable validation discrimination, smaller training-to-validation AUC difference, and suitability for direct SHAP-based interpretation of predictor contributions. In the internal validation cohort, the final XGBoost model had a calibration intercept of 0.058 (95% *CI*, −0.187 to 0.303), a calibration slope of 1.684 (95% *CI*, 1.304 to 2.064), and a Brier score of 0.143 (Supplementary Table 2). The calibration intercept was close to 0, indicating limited systematic overestimation or underestimation of the average predicted risk. However, the calibration slope and its 95% confidence interval were entirely above the ideal value of 1, demonstrating non-ideal calibration and under-dispersion of predicted probabilities. Thus, the predicted risks were compressed toward the mean, potentially resulting in underestimation of risk among higher-risk women and overestimation of risk among lower-risk women. Although decision curve analysis showed greater net benefit for the XGBoost model than for the treat-all and treat-none strategies across the evaluated threshold range, this finding does not overcome the observed calibration limitation. Recalibration and external validation are required before the predicted probabilities can be used for individual-level clinical decision-making (Figure 3).

### SHAP-based interpretation of the final XGBoost model

**Figure 4:**
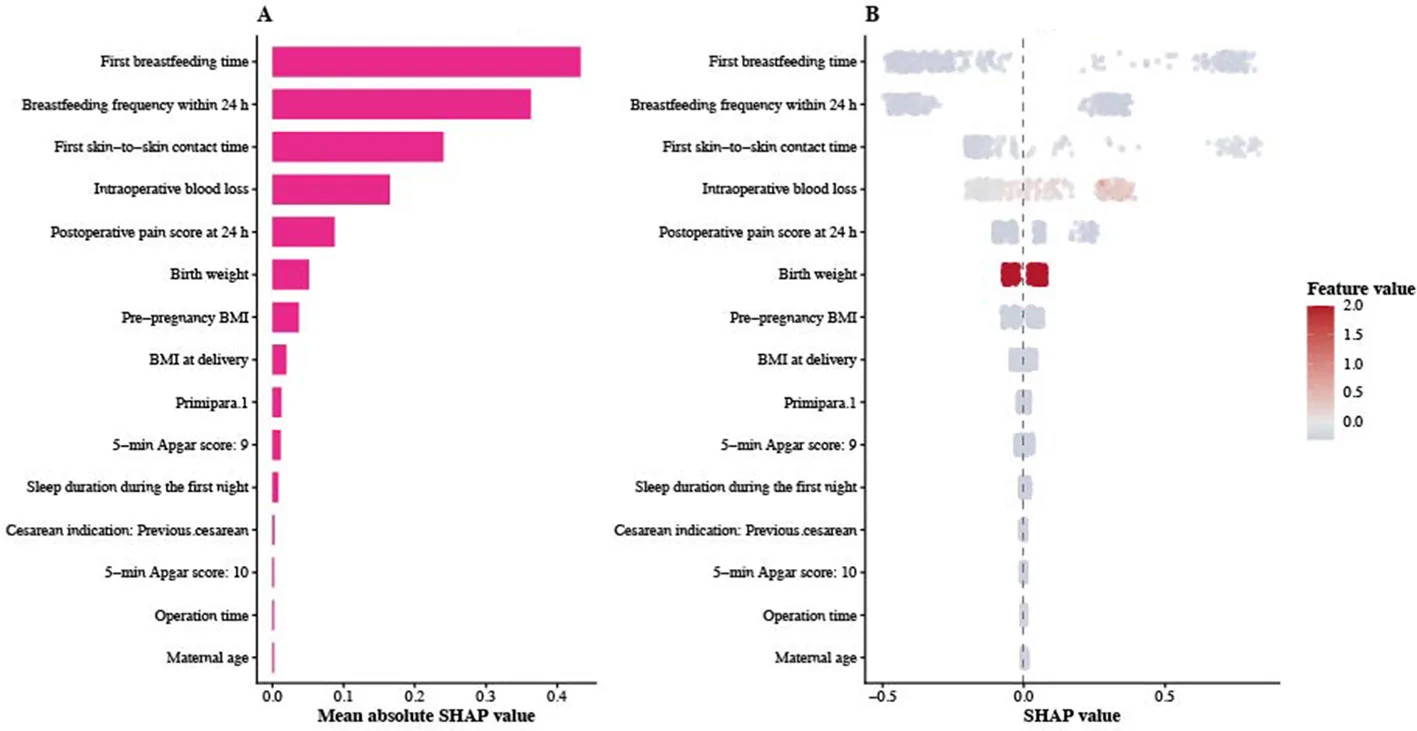
SHAP interpretation of the final XGBoost model. SHAP-based variable importance plot **(A)** and SHAP summary plot **(B)** for the final XGBoost model in the validation cohort. In **(A)**, predictors are ranked according to the mean absolute SHAP value. In **(B)**, each point represents an individual patient, the *x*-axis shows the SHAP value, and the color indicates the relative feature value. Positive SHAP values indicate an increased predicted risk of delayed lactogenesis II, whereas negative SHAP values indicate a decreased predicted risk. SHAP, SHapley Additive exPlanations; XGBoost, extreme gradient boosting.

SHAP analysis was performed to interpret the contribution and direction of predictors in the final XGBoost model. The most influential predictor was first breastfeeding time, followed by breastfeeding frequency within 24 h, first skin-to-skin contact time, intraoperative blood loss, and postoperative pain score at 24 h (Table 4 and Figure 4). Longer time to first breastfeeding, delayed first skin-to-skin contact, greater intraoperative blood loss, higher postoperative pain score, higher birth weight, higher pre-pregnancy BMI, higher BMI at delivery, and primiparity were associated with an increased predicted risk of delayed lactogenesis II. In contrast, higher breastfeeding frequency within 24 h and longer sleep duration during the first postpartum night were associated with a lower predicted risk. The SHAP summary plot further demonstrated that first-24-h postpartum nursing process variables contributed more strongly to the model output than most demographic or routine obstetric variables. These findings suggest that early postpartum nursing process variables may serve as clinically interpretable risk markers for identifying women who remain at increased risk of delayed lactogenesis II after the first 24 h postpartum (Table 4 and Figure 4).

**Table 4:** Key predictors and SHAP-based importance ranking in the final XGBoost model.

Rank	Predictor	Mean absolute SHAP value	Relative importance (%)	Direction of association
1	First breastfeeding time	0.4329	100.0	Higher value associated with higher predicted risk
2	Breastfeeding frequency within 24 h	0.3628	83.8	Higher value associated with lower predicted risk
3	First skin-to-skin contact time	0.2400	55.4	Higher value associated with higher predicted risk
4	Intraoperative blood loss	0.1647	38.0	Higher value associated with higher predicted risk
5	Postoperative pain score at 24 h	0.0876	20.2	Higher value associated with higher predicted risk
6	Birth weight	0.0511	11.8	Higher value associated with higher predicted risk
7	Pre-pregnancy BMI	0.0373	8.6	Higher value associated with higher predicted risk
8	BMI at delivery	0.0198	4.6	Higher value associated with higher predicted risk
9	Primipara	0.0128	3.0	Higher value associated with higher predicted risk
10	Five-min Apgar score of 9	0.0114	2.6	Higher value associated with higher predicted risk
11	Sleep duration during the first night	0.0079	1.8	Higher value associated with lower predicted risk
12	Cesarean indication: previous cesarean delivery	0.0027	0.6	Higher value associated with lower predicted risk
13	Five-min Apgar score of 10	0.0023	0.5	Higher value associated with lower predicted risk
14	Operation time	0.0022	0.5	Higher value associated with higher predicted risk
15	Maternal age	0.0020	0.5	Higher value associated with higher predicted risk

BMI, body mass index; SHAP, SHapley Additive exPlanations; XGBoost, extreme gradient boosting. SHAP values were calculated in the validation cohort using the final XGBoost model. Predictors were ranked according to the mean absolute SHAP value, which reflects the average contribution of each predictor to model output. Relative importance was scaled to the highest mean absolute SHAP value. The direction of association was estimated according to the relationship between predictor values and SHAP values; a positive direction indicates that higher values or the presence of the characteristic tended to increase the predicted risk of delayed lactogenesis II. Because pre-pregnancy BMI and BMI at delivery were strongly correlated, their individual mean absolute SHAP values may reflect shared or redistributed predictive information and should not be interpreted as independent effect estimates.

### Exploratory risk stratification

In the internal validation cohort, the exploratory risk-stratification scheme classified 111 women as low risk, 175 as moderate risk, and 100 as high risk. Delayed lactogenesis II occurred in 6 women in the low-risk group, 30 women in the moderate-risk group, and 62 women in the high-risk group, corresponding to observed event rates of 5.4%, 17.1%, and 62.0%, respectively. The observed incidence increased significantly across the three risk groups (*P* < 0.001; Supplementary Table 4). These findings demonstrate that the model could distinguish groups with markedly different observed risks. However, the proposed cutoffs were exploratory and should not be interpreted as validated thresholds for initiating or withholding clinical interventions.

### Landmark-restricted sensitivity analysis

Of the 1,286 women included in the primary analysis, 71 (5.5%) had already experienced lactogenesis II by the 24-h assessment and were therefore no longer at risk of delayed lactogenesis II at the prediction landmark. After excluding these women, the landmark-restricted cohort comprised 1,215 women, including 326 who developed delayed lactogenesis II by 72 h postpartum. The original cohort assignments were retained, resulting in a restricted training cohort of 851 women, including 228 outcome events, and a restricted internal validation cohort of 364 women, including 98 outcome events. When the complete modeling procedure was repeated in the restricted at-risk population, the XGBoost model achieved a validation AUC of 0.817. The calibration intercept was 0.041, the calibration slope was 1.592, and the Brier score was 0.150. First breastfeeding time, breastfeeding frequency within 24 h, first skin-to-skin contact time, intraoperative blood loss, and postoperative pain score at 24 h remained the five leading predictors. These results were broadly consistent with those of the primary analysis, although calibration remained non-ideal, and external validation and recalibration are required before clinical application (Supplementary Table 6).

## Discussion

In this retrospective cohort study of 1,286 women who underwent cesarean delivery, we developed and internally validated five dynamic 24-h postpartum landmark prediction models for delayed lactogenesis II by 72 h after delivery using routinely collected clinical, perioperative, neonatal, and first-24-h nursing process variables. The overall incidence of delayed lactogenesis II was 25.3%, indicating that delayed onset of copious milk production remains a common early postpartum problem in women after cesarean delivery. In the validation cohort, all five models demonstrated acceptable discrimination, with AUC values ranging from 0.816 to 0.836. Although SVM achieved the numerically highest validation AUC, its discrimination did not differ significantly from that of XGBoost according to the paired DeLong test. XGBoost was retained as the final interpretable model because it showed a smaller training-to-validation AUC difference and enabled direct SHAP-based characterization of predictor importance and directionality. SHAP analysis identified first breastfeeding time, breastfeeding frequency within 24 h, first skin-to-skin contact time, intraoperative blood loss, and postoperative pain score at 24 h as the most important predictors. Importantly, these findings should be interpreted within the 24-h postpartum landmark framework: the model is intended for early postpartum risk reassessment after initial nursing observation, rather than for preoperative or immediate post-delivery prediction. In the primary analysis, 71 women who had already experienced lactogenesis II by the 24-h assessment were retained because the outcome was initially operationalized according to lactogenesis status by 72 h postpartum. Because these women were no longer at risk at the prediction landmark, we additionally repeated the analysis after excluding them. The landmark-restricted sensitivity analysis yielded similar discrimination and the same five leading predictors, indicating that the principal findings were not materially driven by the inclusion of women with early lactogenesis II onset.

The observed incidence of delayed lactogenesis II in our cohort is clinically plausible and consistent with the concept that cesarean delivery is a high-risk setting for early lactation difficulty. Previous prospective studies have shown that delayed lactogenesis is associated with suboptimal breastfeeding practices and reduced breastfeeding success, supporting its relevance as an early postpartum outcome (17). Studies among primiparous mothers and other postpartum populations have also demonstrated that delayed lactogenesis II is influenced by both maternal factors and early breastfeeding-related behaviors (18). Our study extends this literature by focusing specifically on women after cesarean delivery and by incorporating first-24-h nursing process variables into a machine learning framework for dynamic postpartum risk stratification.

The most influential predictor in the final XGBoost model was first breastfeeding time. Longer time to first breastfeeding was associated with higher predicted risk of delayed lactogenesis II. This finding is clinically meaningful because early breastfeeding initiation is closely related to nipple–areolar stimulation, maternal oxytocin and prolactin release, milk removal, and maternal confidence in breastfeeding. In the context of cesarean delivery, delayed first breastfeeding may also reflect postoperative pain, delayed recovery from anesthesia, limited maternal mobility, mother–infant separation, or insufficient early breastfeeding assistance. Therefore, within this prediction model, first breastfeeding time should be interpreted as a dynamic early postpartum risk marker that may reflect the feasibility and context of initial breastfeeding support. Because later first breastfeeding may also result from maternal or neonatal difficulties that subsequently contribute to delayed lactogenesis II, the present association should not be interpreted as evidence that breastfeeding timing independently causes the outcome.

Breastfeeding frequency within the first 24 h was the second most important predictor and showed a protective direction in SHAP analysis. Women with more frequent breastfeeding episodes during the first postpartum day had lower predicted risk of delayed lactogenesis II. This is physiologically plausible because frequent suckling and milk removal are important for stimulating and maintaining milk production. In contrast, low breastfeeding frequency may reflect poor latch, maternal fatigue, pain, neonatal observation, mother–infant separation, or early feeding difficulties. Previous cohort evidence suggests that delayed lactogenesis is closely related to early breastfeeding practices, reinforcing the importance of frequent and effective breastfeeding support during the early postpartum period (19). In the present model, breastfeeding frequency within 24 h is best understood as a dynamic nursing-process risk marker that helps identify women who may remain at increased risk after the first postpartum day. A low breastfeeding frequency may be both a component of the early care process and a consequence of emerging maternal or neonatal feeding difficulties; therefore, its SHAP contribution does not establish a causal protective effect of increasing feeding frequency.

First skin-to-skin contact time was another major predictor. Delayed skin-to-skin contact was associated with increased predicted risk of delayed lactogenesis II. Early skin-to-skin contact facilitates neonatal feeding readiness, supports early breastfeeding initiation, improves maternal–infant interaction, and may reduce maternal stress. Randomized and prospective studies after cesarean delivery have shown that longer or earlier skin-to-skin contact is associated with improved early breastfeeding initiation and exclusivity during hospitalization (20, 21). However, implementation after cesarean delivery may be affected by operating room workflow, maternal monitoring, anesthesia recovery, staff availability, and neonatal condition. Our findings support the value of recording skin-to-skin contact as part of early postpartum nursing assessment and suggest that delayed skin-to-skin contact may help identify women who require closer breastfeeding support.

Intraoperative blood loss was also an important contributor to predicted risk. Greater blood loss may reflect more complex surgery, greater maternal physiological stress, delayed postoperative recovery, or increased fatigue, all of which may interfere with early breastfeeding behaviors. In addition, blood loss and hemodynamic instability may affect maternal endocrine and metabolic adaptation during the immediate postpartum period. Although severe postpartum hemorrhage has been recognized as a factor that may compromise lactation, our findings suggest that routinely recorded intraoperative blood loss may also carry predictive information within a general cesarean-delivery cohort. Women with higher intraoperative blood loss may therefore warrant closer postpartum lactation assessment, assisted breastfeeding positioning, and more intensive monitoring of neonatal intake.

Postoperative pain score at 24 h was among the top five predictors. Pain after cesarean delivery can interfere with breastfeeding by limiting maternal mobility, reducing the ability to hold the infant comfortably, delaying ambulation, and making breastfeeding positioning more difficult. Several studies have reported that post-cesarean pain affects early breastfeeding and infant care, highlighting the importance of adequate postoperative analgesia and nursing support (22, 23). More recent evidence also suggests that higher acute post-cesarean pain is associated with poorer in-hospital breastfeeding outcomes (24). Therefore, pain assessment should not be regarded only as a postoperative recovery indicator but also as a relevant component of early postpartum breastfeeding risk reassessment after cesarean delivery.

Pre-pregnancy BMI, BMI at delivery, primiparity, and birth weight also contributed to the model output, although they were less influential than the leading first-24-h nursing process variables. Higher maternal BMI has been associated with delayed lactogenesis and reduced breastfeeding success in previous studies (25, 26). Potential mechanisms include altered prolactin response, insulin resistance, low-grade inflammation, delayed progesterone withdrawal from adipose tissue, and practical difficulties with positioning and latch. In the present cohort, pre-pregnancy BMI and BMI at delivery were strongly correlated, with VIFs of 8.83 and 8.78, respectively. Both were retained because they represent clinically distinct time points; however, their individual SHAP importance values may be shared or redistributed because of their high correlation. Consequently, their separate SHAP rankings should not be interpreted as evidence that one BMI measure has an independent or stronger causal contribution than the other. Primiparity may increase predicted risk because first-time mothers often have less breastfeeding experience, greater uncertainty, and greater dependence on professional support. These findings indicate that baseline maternal and neonatal characteristics remain relevant but should be interpreted jointly with dynamic postpartum nursing indicators.

The choice of XGBoost as the final interpretable model requires careful consideration because SVM achieved the numerically highest validation AUC. The absolute validation AUC difference between SVM and XGBoost was only 0.013, and the paired DeLong test showed that this difference was not statistically significant (95% *CI*, −0.019 to 0.045; *P* = 0.421). In addition, XGBoost showed a smaller training-to-validation AUC difference than SVM (0.049 vs. 0.097), suggesting less apparent performance optimism across the random split. We therefore did not interpret the small numerical difference in validation AUC as evidence that SVM was statistically superior. Instead, XGBoost was retained on the basis of an overall balance among validation discrimination, apparent stability, and interpretability. Gradient boosting methods are well suited to structured clinical data because they can capture non-linear relationships and interactions between predictors while maintaining strong predictive performance (27). In addition, SHAP-based analysis permitted transparent assessment of global predictor importance, predictor directionality, and individual-level contributions (28). This feature was particularly relevant to the intended nursing application, in which predicted risk should be accompanied by clinically recognizable information that may guide further postpartum assessment and support.

Model interpretability is especially important in postpartum nursing practice. A black-box risk score may be difficult for nurses to trust or translate into action. In contrast, SHAP analysis showed that the most influential predictors were clinically recognizable first-24-h care-process variables. This makes the model more useful for practice because it does not simply classify women as high or low risk; it also highlights clinical features that may guide further assessment. For example, a woman with delayed first breastfeeding, low breastfeeding frequency, delayed skin-to-skin contact, greater blood loss, and higher pain score could be prioritized for timely lactation consultation, assisted positioning, analgesic optimization, structured breastfeeding monitoring, and closer neonatal intake assessment. However, because this was a retrospective prediction study, these predictors should be interpreted as early postpartum risk markers and potential care-process targets rather than definitive causal determinants of delayed lactogenesis II.

The calibration and decision curve findings provide a more nuanced assessment of the final XGBoost model. Calibration is essential because a model with acceptable discrimination may still produce inaccurate absolute risk estimates when predicted probabilities do not agree with observed risks (29). In the internal validation cohort, the calibration intercept was 0.058, which was close to the ideal value of 0 and indicated limited systematic overestimation or underestimation of the average predicted risk. The Brier score was 0.143. However, the calibration slope was 1.684 (95% *CI*, 1.304–2.064), and the entire confidence interval was above the ideal value of 1. This result demonstrates non-ideal calibration and under-dispersion of predicted probabilities. In other words, the model produced probabilities that were compressed toward the mean, potentially underestimating risk among higher-risk women and overestimating risk among lower-risk women. Decision curve analysis showed that the model provided greater net benefit than the treat-all and treat-none strategies across the evaluated threshold range (30). Nevertheless, decision-curve net benefit does not correct inaccurate individual risk estimates or eliminate the need for appropriate calibration. Therefore, the model's absolute risk estimates should not currently be used for individual-level clinical decision-making. External validation and recalibration are required before clinical application.

The main clinical implication of this study is that delayed lactogenesis II after cesarean delivery may be approached as a dynamic postpartum care problem rather than solely as a consequence of fixed maternal characteristics. The exploratory risk-stratification analysis illustrated one possible application of the model: the observed event rates were 5.4%, 17.1%, and 62.0% in the low-, moderate-, and high-risk groups, respectively. If confirmed in future validation studies, such stratification could support graduated nursing assessment, with routine breastfeeding support for women at low risk, enhanced breastfeeding and latch assessment for those at moderate risk, and early lactation consultation and closer maternal–neonatal monitoring for those at high risk. The strongest predictors—first breastfeeding time, breastfeeding frequency within 24 h, skin-to-skin contact, blood loss, and pain—may help clinicians understand why an individual woman receives a higher predicted risk. However, the proposed cutoffs are exploratory, and the model does not establish that modifying any predictor will prevent delayed lactogenesis II. Because the model has undergone only single-center internal validation and showed non-ideal calibration, external validation and recalibration are required before it can support individual-level clinical decision-making.

Further validation will follow a staged approach. First, temporal validation is planned using consecutive women undergoing cesarean delivery at the same institution after the end of the current study period. The current model specification, preprocessing procedure, and proposed risk thresholds will be frozen before this evaluation to minimize optimistic bias. Second, multicenter external validation is planned in hospitals with different patient populations, perioperative practices, and postpartum breastfeeding-support pathways. These studies will evaluate discrimination, calibration intercept, calibration slope, Brier score, decision-curve net benefit, and the observed event rates within each risk group. Recalibration of the intercept and slope will be performed during external validation before consideration of more extensive model updating. Prospective evaluation will then be required to determine whether model-guided nursing support improves lactation and breastfeeding outcomes.

This study has several strengths. First, the sample size was relatively large for a single-center lactation prediction study, with 1,286 women and 326 outcome events. Second, the study focused on women after cesarean delivery, a clinically important population with increased risk of early breastfeeding difficulty. Third, the model incorporated routinely recorded first-24-h nursing process variables, which are often underused in prediction studies but are directly relevant to care delivery. Fourth, model development combined repeated stratified five-fold cross-validation for hyperparameter selection with an independent holdout cohort for final internal validation, and model performance was evaluated in terms of discrimination, calibration, and clinical utility. Finally, SHAP analysis improved model transparency and helped identify clinically interpretable early postpartum risk markers.

Several limitations should be acknowledged. First, this was a single-center retrospective study, and the model requires external validation in other hospitals and regions before clinical implementation. External validation is essential because prediction model performance may decline when applied to populations that differ from the development cohort (31). The primary analysis used complete cases, and 32 women (2.2% of those initially screened) were excluded because of incomplete key predictor data. Although this proportion was low, complete-case exclusion may still introduce selection bias if missingness was related to unmeasured maternal, neonatal, or care-process characteristics. Second, although delayed lactogenesis II was assessed at predefined time points using a standardized maternal question, supporting indicators, and a departmental nursing assessment form, the outcome remained partly dependent on maternal perception and routine clinical documentation. Formal inter-rater reliability was not assessed during routine care, and some degree of non-differential outcome misclassification cannot be excluded. Moreover, the retrospective data extractors were not formally blinded to the 72-h lactogenesis outcome. Although predictor definitions were prespecified and predictor ascertainment was restricted to structured or time-stamped information documented by the 24-h landmark, some risk of observer-related information bias cannot be completely excluded. Third, some potentially important variables were not fully available in routine records, including detailed breastfeeding self-efficacy, maternal psychological status, professional lactation consultation intensity, intraoperative fluid therapy, detailed analgesic regimens, and neonatal sucking effectiveness. Fourth, although the 24-h landmark and exclusion of post-landmark outcome-related variables reduced the risk of information leakage, they could not completely eliminate reverse causation. First breastfeeding time and breastfeeding frequency within 24 h may reflect both early care processes and emerging maternal or neonatal feeding difficulties. Moreover, SHAP values quantify contributions to model predictions but do not establish causal effects. In addition, pre-pregnancy BMI and BMI at delivery were highly correlated. Although this does not prevent their use in a tree-based prediction model, correlated features may divide or redistribute SHAP importance. Their individual importance rankings should therefore be interpreted cautiously and should not be regarded as estimates of independent effects. These variables should therefore be interpreted as dynamic risk markers rather than modifiable causal determinants, and the present findings cannot establish that changing breastfeeding timing or frequency would reduce the occurrence of delayed lactogenesis II. Fifth, the model should be interpreted as a 24-h postpartum dynamic reassessment tool rather than a preoperative or immediate post-delivery prediction model. In the primary analysis, 71 women with documented lactogenesis II onset by the 24-h assessment were retained because the outcome was initially operationalized according to lactogenesis status by 72 h postpartum. These women were no longer at risk of delayed lactogenesis II at the 24-h prediction landmark, and their inclusion meant that the primary cohort did not strictly correspond to the population remaining at risk at the time of prediction. We therefore performed a landmark-restricted sensitivity analysis after excluding these women. The restricted analysis produced similar discrimination and retained the same leading predictors, suggesting that the principal findings were not materially driven by their inclusion. Nevertheless, the restricted cohort more accurately represents the intended target population, and future temporal and external validation should be conducted exclusively among women who have not experienced lactogenesis II by the 24-h landmark. Several important predictors, including breastfeeding frequency within 24 h and postoperative pain score at 24 h, were measured during the early postpartum period. Therefore, the model is intended for identifying women who remain at increased risk after initial postpartum observation, not for risk prediction before the initiation of postpartum nursing care. In addition, although the calibration intercept was close to 0, the calibration slope was 1.684 (95% *CI*, 1.304–2.064), indicating non-ideal calibration and under-dispersion of predicted probabilities. Because the predicted probabilities were compressed toward the mean, the model may underestimate risk among higher-risk women and overestimate risk among lower-risk women. Consequently, external validation and recalibration are required before the absolute risk estimates can be used for individual-level clinical decision-making. Furthermore, the proposed three-level risk stratification was developed as an exploratory illustration using the same internal validation cohort. Its cutoffs and observed event rates may not generalize to other hospitals or postpartum care settings. Therefore, this stratification should not be used to guide clinical interventions until it has undergone temporal and multicenter external validation. Finally, although repeated stratified cross-validation was used within the training cohort and the internal validation cohort was kept independent from model development, both cohorts originated from the same single-center population. The resulting performance estimates may therefore remain optimistic compared with temporal or multicenter external validation.

In conclusion, this study developed and internally validated a dynamic 24-h postpartum landmark interpretable machine learning model for predicting delayed lactogenesis II by 72 h after cesarean delivery. The final XGBoost model showed acceptable discrimination; however, its calibration was non-ideal, with predicted probabilities compressed toward the mean. SHAP analysis identified first breastfeeding time, breastfeeding frequency within 24 h, first skin-to-skin contact time, intraoperative blood loss, and postoperative pain score at 24 h as the most important predictors. These findings highlight the predictive value of first-24-h nursing process variables and suggest that early postpartum risk reassessment may help identify women who warrant intensified breastfeeding assessment and individualized nursing support after cesarean delivery. These predictors should be interpreted as risk markers rather than causal determinants. The exploratory risk categories require temporal and multicenter external validation, and prospective studies are needed to determine whether model-guided nursing interventions improve lactogenesis and exclusive breastfeeding outcomes. Because the model has undergone only single-center internal validation, it should not be used to estimate individual risk or guide individual-level clinical decisions until external validation and appropriate recalibration have been completed.

## Data Availability

The original contributions presented in the study are included in the article/Supplementary material, further inquiries can be directed to the corresponding author.
