# Supplementary material for: Development and internal validation of an interpretable machine learning model using first-24-h postpartum nursing variables to predict delayed lactogenesis II after cesarean delivery
Source: Front Med (Lausanne). 2026 Aug 10;13:1892993. doi: 10.3389/fmed.2026.1892993 (PMC13500598; doi:10.3389/fmed.2026.1892993)
Supplement: Supplementary file 1 [file Table_1.docx]

**Supplementary Table 1.** Hyperparameter s earch ranges and final parameter settings for the five prediction models

| **Model** | **Hyperparameter** | **Search range or candidate values** | **Final selected value** |
| --- | --- | --- | --- |
| Logistic regression | Penalty | Not applicable; unpenalized reference model | None |
| Logistic regression | Maximum iterations | Fixed | 1,000 |
| Random forest | Number of trees | Fixed | 500 |
| Random forest | Variables considered at each split | 3, 5, 7, 9, 11 | 7 |
| Random forest | Minimum terminal-node size | 1, 3, 5, 10 | 5 |
| Random forest | Maximum tree depth | 5, 10, 15, unrestricted | 10 |
| SVM, radial basis function | Cost parameter, C | 0.25, 0.5, 1, 2, 4, 8 | 4 |
| SVM, radial basis function | Kernel width, gamma | 0.005, 0.01, 0.02, 0.05, 0.10 | 0.05 |
| XGBoost | Number of boosting iterations | 100, 200, 300, 500 | 300 |
| XGBoost | Learning rate | 0.01, 0.03, 0.05, 0.10 | 0.03 |
| XGBoost | Maximum tree depth | 2, 3, 4, 5 | 3 |
| XGBoost | Minimum child weight | 1, 3, 5 | 3 |
| XGBoost | Row subsampling fraction | 0.70, 0.80, 1.00 | 0.80 |
| XGBoost | Column subsampling fraction | 0.70, 0.80, 1.00 | 0.80 |
| XGBoost | Minimum loss reduction, gamma | 0, 0.10, 0.20 | 0 |
| XGBoost | L2 regularization, lambda | 0.5, 1, 2 | 1 |
| LightGBM | Number of boosting iterations | 100, 200, 300, 500 | 300 |
| LightGBM | Learning rate | 0.01, 0.03, 0.05, 0.10 | 0.03 |
| LightGBM | Number of leaves | 7, 15, 31, 63 | 15 |
| LightGBM | Maximum tree depth | 3, 5, 7, unrestricted | 5 |
| LightGBM | Minimum observations per leaf | 10, 20, 30, 50 | 20 |
| LightGBM | Feature fraction | 0.70, 0.80, 1.00 | 0.80 |
| LightGBM | Bagging fraction | 0.70, 0.80, 1.00 | 0.80 |
| LightGBM | L2 regularization | 0, 0.5, 1, 2 | 1 |

**Note:** Hyperparameter tuning was performed exclusively in the training cohort using repeated stratified five-fold cross-validation with five repetitions. The mean cross-validated area under the receiver operating characteristic curve was used as the optimization criterion. Logistic regression was included as an unpenalized conventional reference model and therefore did not undergo hyperparameter tuning. The number of trees in the random forest model was fixed at 500 to stabilize predicted probabilities. After the optimal parameter combination had been selected, each model was refitted using the complete training cohort and evaluated once in the untouched internal validation cohort. A fixed random seed of 2026 was used to ensure reproducibility. AUC, area under the receiver operating characteristic curve; SVM, support vector machine; XGBoost, extreme gradient boosting; LightGBM, light gradient boosting machine.

**Supplementary Table 2.** Quantitative calibration measures of the final XGBoost model in the internal validation cohort

| **Calibration measure** | **Estimate** | **95% CI** | **Ideal value** | **Interpretation** |
| --- | --- | --- | --- | --- |
| Calibration intercept | 0.058 | −0.187 to 0.303 | 0 | Limited systematic overestimation or underestimation of average predicted risk |
| Calibration slope | 1.684 | 1.304 to 2.064 | 1 | Non-ideal calibration; predicted probabilities were under-dispersed and compressed toward the mean |
| Brier score | 0.143 | Not applicable | 0 | Mean squared prediction error; lower values indicate better probabilistic accuracy |

**Note:** Calibration measures were calculated in the internal validation cohort (n = 386). The calibration intercept assesses systematic overestimation or underestimation of the average predicted risk, with an ideal value of 0. The calibration slope assesses the spread of predicted probabilities, with an ideal value of 1. The observed calibration slope of 1.684 (95% CI, 1.304–2.064), with the entire confidence interval above 1, indicates non-ideal calibration and under-dispersion of predicted probabilities. Thus, the predicted risks were compressed toward the mean, potentially resulting in underestimation of risk among higher-risk women and overestimation of risk among lower-risk women. The Brier score is the mean squared difference between predicted probabilities and observed outcomes and ranges from 0 to 1, with lower values indicating better probabilistic accuracy. External validation and recalibration are required before the model can be used to provide individual-level absolute risk estimates or support individual-level clinical decision-making. CI, confidence interval; XGBoost, extreme gradient boosting.

**Supplementary Table 3.** Correlation and multicollinearity assessment of selected continuous predictors in the training cohort

| **Predictor** | **VIF** | **Interpretation** |
| --- | --- | --- |
| Pre-pregnancy BMI | 8.83 | Substantial collinearity with BMI at delivery |
| BMI at delivery | 8.78 | Substantial collinearity with pre-pregnancy BMI |
| First skin-to-skin contact time | 2.50 | No important multicollinearity |
| First breastfeeding time | 1.33 | No important multicollinearity |
| Intraoperative blood loss | 1.21 | No important multicollinearity |
| Postoperative pain score at 24 h | 1.17 | No important multicollinearity |
| Breastfeeding frequency within 24 h | 1.14 | No important multicollinearity |
| Sleep duration during the first night | 1.12 | No important multicollinearity |
| Birth weight | 1.08 | No important multicollinearity |
| Maternal age | 1.06 | No important multicollinearity |
| Operation time | 1.06 | No important multicollinearity |
| Gestational age | 1.04 | No important multicollinearity |

**Note:** Multicollinearity was assessed in the training cohort. Pre-pregnancy BMI and BMI at delivery were strongly correlated (Spearman’s $r_{s}=0.938$, P<0.001; Pearson’s r=0.938, P<0.001). A VIF ≥5 was considered indicative of potentially important collinearity, and a VIF ≥10 was considered indicative of severe multicollinearity. BMI, body mass index; VIF, variance inflation factor.

**Supplementary Table 4.** Exploratory risk stratification in the internal validation cohort

| **Risk group** | **Predicted-risk range** | **No. of women** | **Delayed lactogenesis II, n** | **Observed event rate** |
| --- | --- | --- | --- | --- |
| Low risk | <0.15 | 111 | 6 | 5.4% |
| Moderate risk | 0.15 to <0.30 | 175 | 30 | 17.1% |
| High risk | ≥0.30 | 100 | 62 | 62.0% |

**Note:** Risk groups were defined using the predicted probabilities generated by the final XGBoost model in the internal validation cohort. Differences in observed event proportions across the three groups were assessed using Pearson’s chi-square test (P<0.001). The proposed cutoffs are exploratory and are intended to illustrate potential clinical risk stratification; they have not been externally validated and should not be interpreted as definitive intervention thresholds. XGBoost, extreme gradient boosting.

**Supplementary Table 5.** Summary of outcome and predictor-data completeness

| **Data-completeness category** | **No. of women** | **Percentage of initially screened cohort** |
| --- | --- | --- |
| Initially screened | 1,472 | 100.0% |
| Missing 72-hour outcome data | 9 | 0.6% |
| Missing data for ≥1 key candidate predictor | 32 | 2.2% |
| Final complete-case analytic cohort | 1,286 | 87.4% |
| Residual missing outcome values in analytic cohort | 0 | 0.0% |
| Residual missing predictor values in analytic cohort | 0 | 0.0% |

**Note:** Percentages were calculated using the 1,472 initially screened women as the denominator. “Missing data for ≥1 key candidate predictor” refers to participant-level incompleteness; a woman was counted once if any predictor required for model development was missing. The final analytic cohort was based on complete cases, and no single or multiple imputation was performed. Other exclusions were based on the prespecified clinical eligibility criteria and are shown in Figure 1.

**Supplementary Table 6.** Sensitivity analysis restricted to women without lactogenesis II by the 24-hour prediction landmark

| **Measure** | **Primary analysis** | **Landmark-restricted sensitivity analysis** |
| --- | --- | --- |
| Overall sample size | 1,286 | 1,215 |
| Women with delayed lactogenesis II | 326 | 326 |
| Overall event rate | 25.3% | 26.8% |
| Training cohort size | 900 | 851 |
| Training events | 228 | 228 |
| Validation cohort size | 386 | 364 |
| Validation events | 98 | 98 |
| Validation event rate | 25.4% | 26.9% |
| XGBoost validation AUC | 0.823 | 0.817 |
| Calibration intercept | 0.058 | 0.041 |
| Calibration slope | 1.684 | 1.592 |
| Brier score | 0.143 | 0.150 |

**Note:** The landmark-restricted analysis excluded 71 women who had documented onset of lactogenesis II by the 24-hour assessment. The original training and internal validation assignments were retained. The complete model-development procedure was repeated using the same preprocessing, hyperparameter-tuning, threshold-selection and evaluation framework as in the primary analysis. AUC, area under the under the receiver operating characteristic curve; XGBoost, extreme gradient boosting.
